# Problems of Acute Infectious Jaundice in the United States

**Published:** 1919

**Authors:** 


***The Problems of Acute Infectious Jaundice in the United States.*** By M. H. Neill. *Public Health Reports*, Vol. 33, No. 19, May 1918.

This acute infectious disease, characterized at onset by prostration, gastrointestinal symptoms, and later fever and jaundice, is described in detail by Dr. Neill, who takes up its prevalence as a war disease and indicates the points of special interest to sanitarians, with procedure for their laboratory study.

Outbreaks of jaundice have occurred during the war at different times, from Belgium to Gallipoli, and among the troops of every nationality, though the mortality has usually not been high.

It is believed that the causal factor is the " spirochaete icterohemorrhagiæ " first demonstrated in Japan. Noguchi found rats in New York infested with the same organism as that causing jaundice in man, and cross immunity tests proved them the same in that city as in Japan and Europe.

A search of the literature reveals a number of outbreaks of jaundice from time to time in the United States, both in civil and military populations, beginning with the War of 1812. There were 71,691 cases recorded in the war between the States. Some of the descriptions correspond closely to the trench jaundice of the present war. Among the civilian population the disease sometimes attacked men only, at other times children, and again women only, with a high mortality among pregnant women. Sometimes the symptomatology suggested " acute yellow atrophy of the liver ".

There is some evidence that direct contact may have played a part in transmitting the disease, but nothing to prove any occupational or food influence.

It is not proven whether the disease exists as a separate entity or as a manifestation of some other disease, such as typhoid fever or malaria, though its seasonal prevalence occurs when they are at their lowest.

Noguchi states that " the finding of the causative organism of infectious jaundice among wild rats in America, and the identification of this strain with those found in Asia and Europe seems to be particularly important in revealing a latent danger to which we have been constantly exposed but from which we escape as long as sanitary conditions are not disturbed by untoward events ". These organisms live in the kidneys of rats and mice without injury to the animal. They were demonstrated in the rats taken from the trenches in which disease had appeared among the troops. The theory is that the organism is excreted in the urine of the rat and, in a favorable environment, may live long enough to infect a human by means of the skin or mouth.

Laboratory methods successfully employed in examination of human beings :

(1) Examination of blood films,

(2) Examination of blood by dark field illumination,

(3) Injection of blood into the peritoneal cavity of a guinea pig.

Of these the inoculation is most valuable and should always be done when the patient is seen early.

On opening the skin of the abdomen, one is struck with the widespread hemorrhages beneath the skin, especially about the axillary and inguinal lymph-nodes. Post-peritoneal hemorrhages are frequent and abundant. The kidney is also frequently the seat of marked effusion of blood. In the lungs there are numerous sharply defined hemorrhagic foci. The liver shows exudation of poly-morphonuclear leucocytes, and the kidney an acute exudative nephritis.

(4) Microscopic examination of urine, which requires familiarity with the organism under discussion, and should be in expert hands.

(5) The injection of urinary sediment into the peritoneal cavity of a guinea-pig; this has been followed by positive results and should be regularly practiced.

(6) Examination of tissues obtained at necropsy by the method of Levaditi, which may demonstrate the spirochete in viscera, especially the kidneys.

In examining rats, after being killed the kidneys are removed, using care not to contaminate them. They are emulsified and injected into the peritoneal cavity, using a guinea-pig for each rat, and watching it for at least two weeks. If the organism is present, the pig will become ill, slightly jaundiced, and will collapse and die in about ten days.

The author appends a bibliography of the literature concerning this organism and its effects on man and animals.

